# On Somnambulism

**Published:** 1856-01-01

**Authors:** 


### Art. II.—ON SOMNAMBULISM.

In common with all animals which possess well-defined sensuous relations with the external world, man exists in two distinct, and, so far as the organs of these relations are involved, opposed conditions—one of waking and one of sleep, labour and repose alternating. Under certain limitations, this alternation appears to be a general law of organization, more or less modified according to the varying complexity of the functions of life. It is true that in sleep only the animal or relational functions are at rest; the repose of the tissues concerned in vegetative life is of much shorter duration, action and rest recurring every instant. It is in accordance with the same principles that we find the amount and regularity of sleep in great measure proportionate to the development of relational life. In the higher carnivorous vertebrata, where the muscular and nervous tissues are at the maximum development, sleep is much more required than in those of lower type where the nutritive functions appear predominant; and in those lowest forms of organic existence which still appear to have some trace of animal nature, but whose chief and entire function appears to be assimilative, we have no evidence of the occurrence of the phenomenon at all. As might be expected, it is in man, where the balance of the two classes of functions is most evident, and where the operations are still more complicated by the superaddition of an intellectual nature, that the periodical recurrence of repose is most marked, and its regularity most essential to the well-being of the individual.

It will materially assist our investigation into some of the interesting phenomena involved in our subject, if we briefly examine the points of contrast between these two opposed conditions, as well as the points of resemblance, and those states in which they appear to trespass upon each other's domains.

What are the characteristics of a healthy waking man, *mens*

*sana in corpore sano?*—As the basis of all his knowledge, and of all his actions, there is a profound conviction and consciousness of distinct existence and personality, a strong intuitive and undefinable, yet irrefragable, sense of the unchanging "I." (It is necessary to mention this fundamental truth, because in dreaming, and certain forms of insanity, it is very frequently utterly lost from the mind.) This consciousness is modified and intensified by the evidence of the senses—these respond instantaneously and accurately to their own appropriate stimuli, the eye to the undulations of light, the ear to the vibrations of sound, and so on with the other senses, none of which can supply the place of another; nor is the general sense of touch ever capable of being exalted to the condition of a special sense. But not only do these organs take cognizance of the external world and its phenomena, but the mind receives the impressions from them, and is prepared at once to exercise upon them its various functions; memory, imagination, fancy, comparison, judgment, calculation, all these, and all other faculties into which metaphysicians have dissected the Divine spark, are either in activity, or ready to be so, at the command of the will. Finally, the muscular system obeys accurately the mandates of the will.

So far as to the positive phenomena—but the negative indications of health and wakefulness are not less important for our purposes. These may be briefly summed up in a few words—*complete unconsciousness of all organic or vegetative processes.* And during this time a waste, both of substance and of vital energy is going on, which requires the periodical return of sleep for its repair, the phenomena of which condition we have now to notice.

> "Somne, quies rerum, placidissime, Somne, Deorum,

> Pax animi, quem cura fugit—"

Thus by negations is sleep invoked by the ancient poet; and certainly sleep in its perfect form is only to be described by negations, with the exception of the continuance of the organic functions, which remain nearly unaffected, or in some cases increased in intensity, as Hippocrates justly observed, *somnus labor visceribus.* Perfect sleep is characterized by a complete and profound unconsciousness of everything, even of existence—the senses are closed against all impressions, the limbs have become relaxed and inactive, even volition, in common with every other faculty of the mind, is in abeyance—phenomena well and elegantly portrayed by Lucretius—

> " ——— ubi est distracta per artus
> Vis animæ—
> Debile fit corpus, languescunt omnia membra,
> Brachia, palpebræque cadunt, poplitesque procumbunt."

Many extraordinary histories are related in illustration of the extent to which insensibility to outward impressions may be carried; one will suffice, as an extreme case. It is quoted by Dr. Carpenter, with tokens of credence from Mr. R. Smith, late senior surgeon to the Bristol Infirmary, under whose observation it occurred. "A travelling man, one winter's evening, laid himself down upon the platform of a lime-kiln, placing his feet, probably numbed with cold, upon the heap of stones, newly put on to burn through the night. Sleep overcame him in this situation; the fire gradually rising and increasing until it ignited the stones upon which his feet were placed. Lulled by the warmth, the man slept on; the fire increased until it burned one foot (which probably was extended over a vent-hole) and part of the leg above the ankle entirely off, consuming that part so effectually that a cinder-like fragment was alone remaining, and still the poor wretch slept on; and in this state was found by the kilnman in the morning." He experienced no pain when he awoke, but he died in hospital about a fortnight afterwards. It appears probable, however, that the atmosphere in this case was charged with carbonic acid, and that the sleep was nearly approaching to, if not altogether identical with, coma.

Sleep is not always, nor even commonly, thus profound; yet, even under its ordinary aspects, it presents such a picture of inactivity as to have been considered by many, both poets and philosophers, as nearly related to death. "Sleep," says Macnish, "is the intermediate state between wakefulness and death." Diogenes is said to have spoken, in his last moments, of death and sleep as brother and sister. Cicero speaks thus of the affinity—*nihil videmus morti tam simile quam somnum;* and Ovid in like manner asks—

"Quid est somnus, gelidæ nisi mortis imago?"

Yet the analogy is much more poetical than true; sleep is as far removed from death as muscular repose is from paralysis. It is probably the normal state of fœtal existence, and throughout life it is the great agent in repairing the ravages of constant molecular changes, and averting the ever-threatening somatic death.

The most usual form of sleep is by no means so profound as that which we have described; some of the functions both animal and intellectual are often at work, and dreaming, with or without accompanying action, is the result. In such a case, a kind of consciousness is restored, yet often with peculiar modifications, one of the most remarkable being the loss of that distinct sense of individuality by which the waking man has been said to be characterized. Imagination and memory are both awake, at times more active than in true wakefulness; but they play strange tricks with each other and with their possessor. He can contemplate his own murder, or attend his own funeral, without any feeling of surprise or awe; he can commit the most fearful crimes without any horror; he sees the most tremendous convulsions of nature and the utter subversion of her ordinary laws without astonishment; he converses with the dead, yet asks not how they have escaped their prison-house; and with the living, whom he knows to be separated from him by seas and continents; and all seems natural and a matter of course. Truly has sleep a thousand sons (*natorum mille suorum*, Ovid).

Such are the ordinary and typical forms of man's two lives—the waking and the sleeping life; yet in this, as in all other instances, nature does nothing by sudden leaps (*nihil per saltum*). As night and day are united by twilight—as the two great divisions of organic existences merge into each other through the scarce distinguishable classes of phytozoa and zoophyta—as the various genera of both sub-kingdoms are united by links very nearly allied to both the neighbours—so waking allies itself to sleep by *abstraction* and *reverie*—so sleep allies itself to waking by *dreaming*, by *sleep-talking*, and by the *sleep-vigil*, commonly called somnambulism. So closely allied are the extreme forms of reverie and of somnambulism—so difficult in some cases is it to state the precise diagnostic marks—that a few remarks on the former will properly precede and illustrate our more especial theme. *Reverie* is a state of the mind in which it wanders to a thousand different subjects independent of volition—the attention cannot be directed to any one point; on the other hand, *abstraction* is characterized by the total absorption of the mind in one subject, the senses taking cognizance *only* of such matters as are connected with the subject under examination. Distinct as are these two conditions in their origin, they are often confounded together; and, indeed, the external phenomena are similar, being summed up in a more or less complete insensibility of surrounding objects or influences. Great students, especially those of the mathematical or physical sciences, are very prone to falling into this state. Sir Isaac Newton is said to have committed many absurdities when thus absorbed; such as taking a lady's finger for a tobacco-stopper. Archimedes, during the siege of Syracuse, was either totally insensible to or regardless of the noisy operations around, and was insisting on finishing the problem on which he was engaged, when he received his death-blow. The mind appears to be in a state of *polarity* with regard to its subject, and only responds to the allied influences. Of its sensibility to questions and remarks on the one subject and no other, advantage may occasionally be taken, both in the waking dream and the dream vigil, to attract the attention and gradually to dissipate the absorbed condition.

An amusing instance is given by Sir Walter Scott, which, though in a romance, is such a life picture, and so perfect an illustration of these remarks, that we may be pardoned for introducing it here. In "St. Ronan's Well," Mr. Touchwood pays a visit to an abstracted clergyman, Mr. Cargill, whom he finds *lost* in Palestine, and whom he cannot recal by any direct address. At length the student raised his head, and spoke as if in soliloquy, "From Acon, Accor, or St. John d'Acre, to Jerusalem, how far?" "Twenty-three miles N.N.W.," answered his visitor, without hesitation. Mr. Cargill expressed no surprise at a question put to himself being answered by the voice of another—it was the tenor of the answer alone which he attended to in his reply. "Ingulphus and Jeffrey Winesauf do not agree in this." The opening for conversation having thus been made, he is gradually led away to other matters.

For further illustrations of this subject, we refer our readers to the seventeenth chapter of "The Philosophy of Sleep," where many amusing and almost incredible accounts are given of extreme abstraction. We should not have dwelt so long on these preliminary topics, but for the light which they seem calculated to throw upon the connexions of the sleep-vigil—a term which we prefer to somnambulism, inasmuch as this latter expresses only the activity of one function—locomotion—which is by no means the most remarkable of the phenomena.

From the state of profound unconsciousness above described, to a condition with difficulty distinguished from waking, we meet with every possible gradation. The faculties one after another awake, till in some cases we meet with perfectly lucid somnambulism. The first step to this is dreaming. Dreams for the most part are incoherent, shadowy resemblances of scenes and ideas before experienced, most frequently in new and grotesque combinations. The reason and judgment are in abeyance,—we reason, and feel satisfied with the justice and propriety of our conclusions; we compose verses which charm us with their elegant cadence, yet if we can recal these processes when we awake, our arguments are nonsense and our lines doggrel. Much more rarely, the dream is not a repetition merely of past thoughts, but is *supplementary* to the day's exercises,—what has been left undone in waking moments is finished—and well finished—in sleep;* compositions which have overtasked the waking mind have been known to be *dreamed out*, and accurately remembered afterwards; new ideas are likewise originated, as was Coleridge's "Kubla Khan," during sleep. Further illus-

* See "Cyc. of Anat. and Physiology"—Art. Sleep. By Dr. Carpenter.

trations may be found in Dr. Good's "Book of Nature," and in "The Philosophy of Sleep," already quoted.

But the dream is occasionally so vivid as to awaken the power of voluntary motion, and the dreamer enacts or speaks his dream. Hence arise gestures, muttering, talking, walking, and the performance of the most complex operations in sleep. It very frequently happens that the dream having been spoken or acted out, the *polarity* of the mind with relation to that subject is exhausted, and the dream is forgotten, so that the sleep-walker is in general quite unconscious not only of the act itself, but of the train of thought which excited or attended it.

Having thus traced the condition of the mind through its successive stages of complete wakefulness, reverie, abstraction, sleep, and dreaming, to a pseudo-waking and active state again, we shall now give a few illustrations of the phenomena of sleep-vigil, beginning with the simplest forms,—viz., where the sleeping acts are mere mechanical repetitions of daily performances; and advancing to those of great intellectual complication, that we may be better prepared by a collection of facts, inductively to ascertain the true and essential nature of the phenomena.

It is those acts which are most habitual by day, that are most frequently re-enacted by night, and these are sometimes of an extraordinary nature. The simplest are those connected with visiting the various scenes of labour. A young man being asleep in the pump-house of the mine in which he worked, rose and walked to the door, against which he leaned some time; then he walked to the engine shaft, and safely descended twenty fathoms, where he was found with his back resting on the ladder. When he had been with difficulty awoke, he was quite at a loss to account for his being there.

Those who ride much on horseback will either do so in their sleep, or will imitate the action, as in a case related by Petrus Diversus, where a young man climbed up, and mounted across the battlements, where he spurred vigorously, and was much alarmed on awaking at the risk he had run.

Others will even swim for a considerable time without awaking, of which there are many instances on record. Dr. Franklin relates that he himself fell asleep whilst floating on his back, and slept for an hour.

In a case related by Macnish, occurring on the coast of Ireland, the sleeper walked through a difficult and dangerous road, nearly two miles, and plunging into the water, had swam a mile and a half, when he was discovered, still fast asleep.

Martinet mentions a case of a watchmaker's assistant who had an attack of somnambulism every fortnight, and in that state, was accustomed to arise and do his usual work with as much accuracy as when awake. Dr. Gall mentions a miller, who every night arose and set his mill working, recollecting nothing of what had passed in the morning. Instances are innumerable of these mechanical employments being carried on in sleep,—it is needless to multiply them—we pass on to cases of a more complex character. In somnambulism the eyes are often shut, and if open, they are evidently not in a state adapted to ordinary vision, as will be described afterwards; yet feats can be performed with safety and accuracy, which the individual would never dare to attempt when awake. An account in illustration we extract from the "Philosophy of Sleep:"—"A story is told of a boy, who dreamed that he got out of bed, and ascended to the summit of an enormous rock, where he found an eagle's nest, which he brought away with him, and placed under his bed. Now the whole of these events actually took place; and what he conceived on waking to be a mere vision, was proved to have had an actual existence, by the nest being found in the precise spot where he imagined he had put it, and by the evidence of spectators, who beheld his perilous adventure. The precipice which he ascended, was of a nature that must have baffled the most expert mountaineer, and such as, at other times, he could never have scaled."

These adventures are not always unattended by danger. Schenkins relates an instance where the somnambulist, in attempting to get out of a window, fell and broke his thigh. A similar accident happened to a Mr. Dubrie, a musician in Bath.

But the phenomena of somnambulism become much more interesting and pregnant with meaning, when the manifestations of activity are more specifically intellectual, and where at the same time the state of the special senses can be made the subject of observation. The senses and the intelligence appear to be closed to ordinary influences, yet susceptible to those connected with the dominant train of thought, sometimes to an almost preternatural extent. We will, however, for the present, proceed with the enumeration of facts, as related by credible writers, leaving our analysis of them to a later period.

Henricus ab Heers relates an instance of a friend of his own, who having been unable to finish some verses to his satisfaction by day, arose in his sleep, finished them, sought out his friends, read the verses to them, and retired to rest again. It was with difficulty that he could be made to believe all that had occurred on the morrow.

A very interesting account of Signor Augustin Forari, a notable somnambulist, is extracted from Muratori, by Dr. Pritchard ("Treatise on Insanity," p. 435), from whom we will borrow some details. The speaker is an eye-witness, Vigneul Marville. "Whilst still asleep," he says, "I examined him with a candle in my hand. He was lying upon his back, and sleeping with open, staring, unmoved eyes. We were told that this was a sure sign that he would walk in his sleep. I felt his hands, and found them extremely cold, and his pulse beat so slowly, that his blood appeared not to circulate. About midnight, Signor Augustin arose, drew aside the bed-clothes with violence, and put on his clothes. I went up to him, and held the light under his eyes. He took no notice of it, though his eyes were open and staring." In this condition he went in and out of several rooms, sought and found different objects, seemed to hear some noises, went to the stable, put the bridle on his horse, but could not find the saddle, mounted, and galloped to the house-door. After performing some other incoherent acts, he went into the billiard-room, and "acted the motions of a player. He then went to a harpsichord, and played a few irregular airs." In about two hours, he went, clothed as he was, threw himself on the bed, and slept many hours.

Louyer-Willermay in the "Dict. des Sciences Médicales," gives a short account of a young man named Negretti, likewise mentioned more at length by Dr. Pritchard. It was first given by an eye-witness, Dr. Pigatti, in the "Journal Étranger," March, 1756. He was a servant, and had walked in his sleep from his eleventh year. He would often repeat in his sleep his accustomed duties of the day, and would carry trays and glasses about, and spread the table for dinner with great accuracy, though Righellini, another eye-witness, asserts that his eyes were always firmly closed. He sometimes carried about a candle, but if a bottle were substituted, it appeared to satisfy him equally well. He sometimes stumbled and struck himself against objects in strange places. His sense of taste appeared to be imperfect, as he would eat cabbage instead of salad, drink water for wine, and having asked for snuff, would take coffee instead.

In other instances, more of the faculties seem to have been awake. Castelli, whose case is related by an Italian physician, was found translating Italian into French in his sleep, and looking for the words in a dictionary. When his candle was extinguished, he could not proceed till he had relighted it. When he was addressed on any subject on which he was occupied, he answered rationally, but heard nothing which was not in conformity with that train of thought. The sense of sight appeared similarly affected. For further details and comments upon these cases, we refer the reader to the work by M. Bertrand which is mentioned at the head of this paper.

The next class of cases will indicate a still more singular state of mind and body. For illustration we select two,—the first related by the Archbishop of Bordeaux, concerning a young priest in a catholic seminary. He would rise from his bed, compose, write, and correct sermons. On one occasion, having written the words, *ce divin enfant,* he had effaced the word *divin,* substituting *adorable;* but on re-reading the composition, he found that *ce* would not do to stand before *adorable,* and inserted a *t,* so as to make the word *cet!* Yet, extraordinary to relate, when a card was held between his eyes and the paper, he continued his writing and his corrections as before. He wrote music, too, with exactness; yet all this time, if a piece of paper *exactly the same size* were substituted for that in use, he would go on from the same place, and put his corrections, if required, in the place corresponding to the error in the first paper. If the size varied, he detected the substitution. He asked for certain things, and saw and heard such things, but *only* such things, as bore directly upon the subject of his thoughts. He detected the deceit when water was given to him instead of brandy, which he had asked for. Finally, he knew nothing of all that had transpired when he awoke, but in his next paroxysm he remembered all accurately, and so lived a sort of double life; a phenomenon which we shall find more fully illustrated in other cases, and which we believe to be universal in all the more exalted cases of ecstatic somnambulism.

The next case is extracted from a report made to the Physical Society of Lausanne, by a committee of gentlemen appointed to examine a young man who was accustomed to walk in his sleep. The subject of it was a lad about fourteen years old, of strong constitution, but of great sensibility and irritability. The facts observed were very similar to those just related; the same doubtful indications of the state of the senses—the same consciousness with regard to the objects upon which his thoughts were fixed—and the same insensibility to almost all others. His eyes were for the most part shut, and when writing he continued to do so with the same accuracy, though a piece of thick paper was held between his eyes and the book,—only feeling some little inconvenience from the paper being so near the nose, and probably impeding free respiration. He also forgot everything that had passed during the paroxysm after he awoke, but remembered it all during the next attack. We do not quote the comments of the committee upon this case, as they are of less importance than the facts, for which they seem hardly to account with any probability.

We have now to notice a class of cases which, presenting fewer anomalies in the activity of the organs of sense, are yet more remarkable than the preceding ones, considered as phenomena of sleep, inasmuch as there is very considerable freedom of intercourse with those around; and the condition might naturally be considered as one of perfect wakefulness, but that everything which then happens is forgotten, and only remembered during the next paroxysm.

One of the most remarkable instances of this form of somnambulism is that recorded by Dr. Dyce, of Aberdeen, and quoted by both Dr. Pritchard and Macnish. "The subject of the relation was a girl of sixteen: the first symptom was a propensity to fall asleep in the evening; this was followed by the habit of talking on these occasions, but not incoherently, as sleep-talkers are wont to do. She repeated the occurrences of the day, and sang musical airs, both sacred and profane. Afterwards she became able to answer questions put to her in this state, without being awakened. She dressed the children of the family, still 'dead asleep,' as her mistress termed her state, and once set in order a breakfast-table with her eyes shut." She was taken to church, and appeared much affected by the sermon; but on being questioned, after the fit was over, she denied ever having been to church, but in a subsequent attack, repeated the text and substance of the sermon. Having, by the connivance of a depraved fellow-servant, been ill-treated during one paroxysm, she forgot all about it when awake; but during the next attack, told it to her mother.

A singular and interesting account of a case of spontaneous somnambulism is graphically related by Dr. Carpenter (under whose care the patient was), in the "Cyc. of Anat. and Phys." Art. 'Sleep.' The peculiarity of the case was, the young lady passed into the sleep-walking and talking condition, not as is usual from the sleeping, but from the waking state. She could converse rationally, with one fundamental error or delusion; but she only saw, heard, or understood those objects or ideas which were related to her train of thought; on awaking, all was forgotten, but the same ideas revived and were continued regularly in the next attack. For the very remarkable details, we refer to the article mentioned.

These cases, singular and interesting in themselves, are perhaps still more so, as forming a natural transition and bond of relation between true somnambulism and what has been called double consciousness, a peculiar diplopsychical condition, upon the nature of which little light has hitherto been thrown by either metaphysician or physiologist.

In illustration of this peculiar affection, we shall mention three cases: the first two related by Prof. Silliman, in the "American Journal of Science;" the third, from the "Medical Repository," by Dr. Mitchell.

The subject of the first case was a lady of New England, who became subject to what is called in the report delirium—coming on suddenly, and going off again in the same manner, and leaving the mind quite sound. When in conversation, she would break off in the midst, and begin talking on some subject quite unconnected with the previous one, to which she would not again refer during the continuance of the delirium. "When she became natural again, she would pursue the same conversation in which she had been engaged during the lucid interval, beginning where she had left off—sometimes completing an unfinished story or sentence, or even an unfinished word. When the next delirious paroxysm came on, she would continue the same conversation which she had been pursuing in the preceding paroxysm; so that she appeared as a person might be supposed to do *who had two souls*, each occasionally dormant and occasionally active, and utterly ignorant of what the other was doing."

In quoting this case, Dr. Pritchard very properly remarks:—"It is evident that, although this affection is termed delirium, it was neither that state in the ordinary acceptation of terms, nor any form of madness, but one of *coherent* reverie."

The second case is thus quoted by Dr. Pritchard, from the same source:—"An intelligent lady, in the State of New York, undertook a piece of fine needlework, to which she devoted her time almost constantly for many days. Before its completion she became suddenly delirious, (?) and she continued in that state about seven years. She said not a word during this time about her needlework, but on recovering suddenly from the affection immediately inquired respecting it."

Our next case is so singular and anomalous in its details, that we might hesitate to classify it as one of somnambulism; but we have as yet found no break in our series of phenomena, however strange, arising out of sleep, and the present instance seems so closely allied to those already related that we give it to complete the series. The subject was a young lady, of a good constitution, excellent capacity, and well educated. "Her memory was capacious, and well stored with a copious stock of ideas. Unexpectedly and without any forewarning, she fell into a profound sleep, which continued several hours beyond the ordinary term. On waking, she was discovered to have lost every vestige of acquired knowledge. Her memory was *tabula rasa*—words and things were obliterated and gone. It was found necessary for her to learn everything again. She even acquired, by new efforts, the art of spelling, reading, writing and calculating, and gradually became acquainted with the persons and objects around, like a being for the first time brought into the world. In these exercises she made considerable proficiency. But after a few months another fit of somnolency invaded her. On rousing from it, she found herself restored to the state she was in before the first paroxysm, but was wholly ignorant of every event and occurrence that had befallen her afterwards. The former condition of existence she now calls the Old state, and the latter the New state; and she is as unconscious of her double character as two distinct persons are of their respective natures. For example: in her old state, she possesses all the original knowledge; in her new state, only what she acquired since. If a lady or gentleman be introduced to her in the old state, and *vice versâ* (and so of all other matters), to know them satisfactorily, she must learn them in both states. In the old state, she possesses fine powers of penmanship; while, in the new, she writes a poor awkward hand, having not had time or means to become expert.

During four years and upwards, she has had periodical transitions from one of these states to the other. The alterations are always consequent upon a long and sound sleep. Both the lady and her family are now capable of conducting the affair without embarrassment. By simply knowing whether she is in the old or new state, they regulate the intercourse, and govern themselves accordingly."—("Philosophy of Sleep," note, p. 187.)

All the phenomena occurring in such cases as those already related, appear to be compatible with, at least apparently perfect health. But sleep-walking and sleep-talking occasionally form a part of, or are engrafted upon, hysterical and cataleptic affections —and then we see the proteiform symptoms of hysteria, and the muscular and sensitive derangements of catalepsy added to the sufficiently singular conditions before enumerated. In catalepsy so complicated (and hysteria strongly simulates it frequently), it is usual to see the patient commence and end the paroxysm with the insensible symptoms proper to the disease; whilst the middle part (called the "live fit," in contradistinction to the beginning and end, which are called the "dead fit," in common phrase) is characterized by talking and various actions, evincing a peculiar kind of consciousness and sensibility to certain real or imaginary beings or objects; whilst there is the most profound insensibility to all influences from without. Thus a conversation may be carried on with some imaginary interlocutor, with proper pauses for reply and rejoinder; and with one fundamental error, that conversation may be coherent—yet the sufferer may be pricked or cut without evincing any consciousness; or the most pungent stimuli may be applied to the mouth, nose, or conjunctiva, with the same absence of result. The pages of medical history abound with records of such cases, but we forbear to quote, as we are at present more concerned with somnambulism in its physiological and psychical, rather than its pathological relations.

It will be useful to review briefly the various forms of sleep-vigil found in the foregoing cases, so as to present an analysis of the phenomena. We have met with—

1. *Profound sleep.*—Unconsciousness.
2. *Dreaming.*—Consciousness, memory, fancy, imagination—more rarely judgment and comparison.
3. *Acted dreams.*—All the former faculties enjoying a sort of wakefulness, and at the same time, volition. This class is only intended to include gestures, &c.
4. *True somnambulism.*—Rising from bed, visiting accustomed or unaccustomed scenes, and performing various mechanical acts. Under this head we have seen the individual performing the most dangerous feats, and the command of the muscular system brought to the greatest perfection.
5. *True sleep-vigil.* — Here, in addition to the foregoing phenomena, many acts of the mind are performed, as judgment, synthesis, analysis, &c.; and the senses, though closed to ordinary influences, seem to be brought into some kind of activity. Here begins also double consciousness, as yet extending only to the sleeping state,—that is, the patient knows nothing of the sleeping acts when awake, though he acts when asleep as if upon a consciousness of what has passed when awake, repeating or completing the acts of that condition. But the various paroxysms of sleep-vigil are attended by a continuity of consciousness,—that is, the acts of one are remembered in the next.
6. *Complete double consciousness, or double life.*—A new life, commencing and ending with deep sleep; utter oblivion of everything before passed; this condition alternating with the old life at uncertain intervals, and the paroxysms of indefinite length. This can scarcely be termed somnambulism, but is noticed as being so closely allied by many of its phenomena to that condition.

It will be evident from a careful consideration of these successive conditions, that somnambulism is not, as M. Willermay and many others consider it, an intermediate state between sleep and waking, (*un état intermediaire entre la veille et le sommeil.*)* That in the slighter forms of the affection many of the faculties enjoy a sort of activity, is clear; that in the higher forms of somno-vigil all, or nearly all, are in such a state that it is diffi-

* "Dict. des Sciences Médicales." Art. Somnambulism.

cult to distinguish between these and their waking manifestations, is also evident; but inasmuch as the sleep appears to be more sound than ordinary; as the somnambulist never passes naturally from that condition to one of waking; as there is some danger attendant upon the interruption of that state; and as the mental and bodily activity for the most part is directed only to one class of subjects; it is plain from all this, that this can be no transition stage to the natural waking activity of the functions; in its higher forms also, we are compelled to consider it as something more than the enacting of a dream, however vivid.

What is the condition of the various functions in the somno-vigil?

Noticing first the most obvious, we see the muscular system perfectly under the command of the will,—often more powerful and accurate in its movements than at other times. The condition of the senses is subject to great variety. 1st. The sight. The eyes are sometimes closed, sometimes widely staring and fixed, sometimes agitated by a convulsive movement, the pupils widely dilated or extremely contracted, but in all conditions evidently unfit for ordinary vision, and almost always insensible to any light experimentally thrown upon them. Yet there are often unmistakeable evidences of the recognition of objects—they are often sought for, and found; sometimes with a light, sometimes without; generally the somnambulist finds his way perfectly in the dark, though some will be at great pains to get a light; he will continue to write with the same accuracy as before when an opaque object is held between his eyes and the paper. Dr. Carpenter states that he has seen this in the artificial somnambulism produced by Mr. Braid's hypnotic process. What is the nature of this vision? Can the general sensibility of the surface be in such manner modified as to serve the purposes of sight? It is very improbable, yet such is said to be the case by many of those who practise the various forms of artificial hypnotism. The solitary instance with which we have met, of any somnambulist remembering and relating the phenomena of vision, is to be found in the "Dict. des Sciences Médicales," *sub voce.* The writer, M. Willermay, speaks thus:—"J'ai moi-même, étant très jeune, eprouvé quelques accés légers de somnambulisme, et il me semble que je voyais en dedans de ma tête ce que je voulais écrire sans le secours des yeux."

2. The sense of hearing is also found in very different conditions. Signor Farari heard the slightest noise near him, but apparently misinterpreted it; others are insensible to the loudest noises, but will hold conversations on subjects immediately connected with their specific train of thought.

3 and 4. The smell and the taste present similar contrarieties, sometimes being more sensitive than natural, sometimes less so, and sometimes perverted.

5. The touch is the most active of all the senses, being as much increased in sensibility and accuracy as is the energy of the muscular system; probably much of the information usually obtained by the special senses is acquired through the increased energy of this, or some modification of it.

But what is the proximate cause of all these phenomena, of all this mimicry of waking life? What is the condition of the brain and mind during this state? We have but little knowledge of the physical differences between the brain active and the brain at rest; but we know that a difference does *potentially* exist, and that whilst the brain at rest is in a state of indifference to stimuli, the brain active is in a condition which may not unaptly be called *polarity.* By polarity in general is understood a state of preparedness to respond to special and specific stimuli, and one of indifference to all objects not coming under this category; thus the magnet is polar and responds to steel, at the same time being indifferent to other substances; the charged conductor of an electric machine is polar, and responds to the class of bodies called electric conductors, being indifferent to all others; in all these cases, when the elements of this polarity are brought into relation, the specific phenomena are evolved, and the polarity *resolved* for the moment. Very analogous are the phenomena of the nervous system, each department of which, when active, is in a state of polarity, evincing certain definite and specific acts or feelings when exposed to certain influences. The optic nerve is polar with regard to light, but takes no cognizance of any other agent, and so in great measure with the ear, the taste, and the smell. The sense of touch is polar with regard to objects with which it comes in contact, but takes no impression (or only those of the most obscure character) from those influences which are so powerful upon the other senses. This, then, is a true polarity of the nervous system; and when we consider how analogous the nervous influence is to the electric, in its mode of propagation, and in many of its manifestations (muscular contraction to wit), we cannot be surprised to meet with further analogies in some of the irregularities of polar tension. For instance, an electric jar may be discharged perfectly by the appropriate apparatus, and brought into a state of equilibrium or indifference; yet very shortly, without any recharge, it will be found to be in a partly charged state, and it requires repeated processes ere it is brought finally into a state of rest. The brain, when active, is in a state of tension or polarity; when at rest, as in sound sleep, it is in a state of entire indifference; but in this case, we have the organic processes perpetually continued; and what wonder that the tension of the brain should thereby be often renewed, so as to awake it to some amount of activity; hence all the phenomena of dreaming.

But why is the dream acted?

In the perfectly waking state, any emotion of the mind produces generally some corresponding action of the body, though perhaps slight; in individuals of irritable fibre this is invariable, except it be modified by education. But in a powerfully abstracted state of the mind, when all external influences, except those upon which the mind is employed, are cut off, the body *acts* the thought of the mind with a certainty and precision which frequently enables the bystander to read the train of ideas accurately. In dreaming, where the mind is absorbed utterly in one train of thought, it is but what we might expect, to find the limbs dramatising the pictures presented to the mind; hence the state described in our fourth division, true somnambulism.

But again, why in the higher forms of somno-vigil are the senses in such a peculiar condition?—why so acute with regard to some objects, so dead to others?

We have seen how, in abstraction, the mind gradually excludes all impressions, save those connected with one special train of thought; the student is absorbed in his problem, and hears nothing of the thunder, sees none of the lightning which plays round him,—the most familiar voice or the most unearthly sounds fall alike dead upon his ear. No doubt these sights and sounds produce their proper physical impression upon the organs of sense, but the brain is no longer in a condition to receive them; it is not in a state of polarity to ordinary influences; all its tension has been withdrawn from without, and fixed upon one class of ideas; impressions therefore fall as ineffectively upon it as light might upon the ear, or sound upon the eye. But in sleep and dreaming, there is no necessity to withdraw the attention from one class of ideas to fix them on another; the tension or polarity of the brain is instituted *only* with reference to that particular class which forms the subject of the dream; the senses may be physically impressed by, but the mind does not recognise, any other object, and hence it is not difficult to understand all the apparently anomalous instances of contradictory perception and unconsciousness; the individual is *abstracted*, but still more completely, for obvious reasons, than in his waking moments.

That the mind should, in certain aspects, be even more acute and vigorous than when awake—that tasks should be completed of the most abstruse character, which had baffled the waking energies—all this, received in the light above suggested, will not appear miraculous; all distracting thoughts, all extraneous sources of error, are withdrawn; and the mind, fully awake to this subject, is enabled to devote its concentrated energies to the task.

One mysterious question remains to be asked—What is the nature of the vision which the somnambulist appears to possess? seeing that frequently the eyes are quite closed; and even when not so, they are unadapted to the ordinary mode of receiving visual impressions. Is there a transference of special sensation? is some part of the surface endowed with something analogous to visual faculties? The records of the various forms of hypnotism, vouched for by men of no mean standing or credibility, would appear to favour such a hypothesis; but, in the present stage of our investigation, we feel unprepared to pass a judgment on so vexed a question. On the phenomena of double consciousness we offer no comments, feeling assured that, as yet, our opportunities of observation have been too few and limited to permit of any satisfactory or efficient generalization.

Our subject would be incomplete without some brief notice of artificial somnambulism—called, by various authorities, Hypnotism, Mesmerism, Animal Magnetism, &c. &c.

By certain manipulations, or demands upon the attention in various forms, or many other means practised upon individuals of very mobile, irritable, imaginative, or otherwise excitable temperaments, certain results are produced more or less analogous to the phenomena of somnambulism; but, if any credit be to be attached to the reports, with many of a more wonderful character superadded. The phenomena said to be produced by the magnetizers are as follow:—

1. "A sense of perflation" all over the system, increase of temperature, and what may be termed hyperconsciousness.

2. A state of drowsiness, and partial excitements of the senses.

3. Complete sleep, insensibility to all stimuli—the patient hears and sees nothing, feels no wound or injury to the person—catalepsy.

4. *Somnambulism.*—In this condition, it is averred the patient can barely distinguish by the sight light from darkness, but "the sense of feeling is metamorphosed into something equivalent to sight"—so that colours of objects, and positions of minute bodies are recognised—and reading is accomplished without aid from the eyes. The seat of this supplementary sense is in the epigastrium, the top of the head, and the finger-ends. The patient can see through opaque media, and the sense of hearing becomes preternaturally acute.

Of these phenomena, those included under the first three heads are doubtless to be produced on subjects properly adapted for such production; those of the fourth class, together with numerous others still more wonderful, asserted by the advocates of this system, are properly regarded as fables. The French commission appointed towards the close of the last century to investigate the claims of the mesmerists, which included the celebrated names of Franklin, Bailly, Lavoisier, Jussieu, and many others of equal eminence, reported strongly against them; admitting certain results, but denying the existence of any "magnetic fluid"—asserting that the phenomena were the result of the imagination powerfully influenced—and, finally, that the effects produced might be dangerous, but never useful. As this subject throws no new light upon the phenomena, or relations of *natural* somnambulism, it is unnecessary to pursue it further.

---

